# Tempering Macrophage Plasticity for Controlling SARS-CoV-2 Infection for Managing COVID-19 Disease

**DOI:** 10.3389/fphar.2020.570698

**Published:** 2020-10-16

**Authors:** Devinder Toor, Aklank Jain, Shivani Kalhan, Harmesh Manocha, Vivek Kumar Sharma, Payal Jain, Vishwas Tripathi, Hridayesh Prakash

**Affiliations:** ^1^Amity Institute of Virology and Immunology, Amity University Uttar Pradesh, Noida, India; ^2^Department of Zoology, Central University of Punjab, Bathinda, India; ^3^Department of Pathology, Government Institute of Medical Sciences, Greater Noida, India; ^4^Department of Microbiology, Government Institute of Medical Sciences, Greater Noida, India; ^5^Department of Physiology, Government Institute of Medical Sciences, Greater Noida, India; ^6^Department of Medicine, Government Institute of Medical Sciences, Greater Noida, India; ^7^School of Biotechnology, Gautam Buddha University, Greater Noida, India

**Keywords:** COVID 19 infection, M1/ M2 macrophages, adoptive immunotheraoy, CD8+T cells, Anti viral activity

## Background

The recent outbreak of the COVID-19 has posed an unprecedented challenge to the health care system. Effective and immediate therapeutics are urgently required to control SARS-CoV-2 infection, which is manifested by aberrant immunopathology. Hyper activation of macrophages and neutrophils contributes to acute respiratory distress syndrome, respiratory failure, and subsequent death of COVID-19 cases. Due to the short life span of neutrophils, tempering macrophage plasticity is relevant for the management of COVID-19 cases. In this context, we here propose that either exchange or in situ reprogramming of derailed Th17+ alveolar macrophages/ Slan+ DC with Th1 programmed counterpart would potentially mitigate or abolish pulmonary fibrosis. This is a new pathology, and our knowledge about this disease is limited, therefore, a great deal of work is required before any therapeutics/vaccine could potentially be launched for its eradication. Several recent and compelling studies have proposed several aspects of the pathological basis of the disease (Zhou F. et al., 2020), which enhanced have our understanding of how SARS-CoV-2 is interacting with alveolar and peripheral tissues and causing the death of some infected patients.

## Pathogenesis of COVID-19

SARS-CoV-2 induces pathogenic inflammation, pulmonary fibrosis, acute respiratory distress syndrome (ARDS), and nephropathy which can lead to the death of some infected patients within 2-3 weeks (Zhou F. et al., 2020). Hyper-activation of committed macrophages, in what is termed macrophage activation syndrome (MAS) (Giamarellos-Bourboulis et al., 2020), is associated with ARDS (Konig et al., 2020), which causes respiratory failure and in some cases the death of COVID-19 patients. Autopsy reports and single-cell RNA sequencing (Liao et al., 2020) have revealed the presence of monocyte‐derived *FCN1* ^+^ macrophages and other immune cells like neutrophils (Liao et al., 2020; Yao et al., 2020) in the bronchoalveolar lavage fluid (BALF) of COVID‐19 patients with severe ARDS. However, due to the abundance of macrophages over other immune cells and MAS in COVID 19 patients, these cells seem to be important for pathogenesis.

Transcriptional analysis of BALF further revealed high levels of IFN‐ϒ induced protein-10 (IP‐10) and various chemokines like MCP‐1, CCL2, and CCL7 in BALF of severe COVID-19 patients, which correlated with increased infiltration of monocyte/Macrophages (Xiong et al., 2020) in their lungs. Cytokine profiling of critical COVID-19 patients reflects MAS with high levels of pro-inflammatory cytokines and chemokines such as IL-6, IL-7, Tumour Necrosis Factor (TNF-α), CCL2, CCL3, CXCL10, and IL-2 receptor. This demonstrates dysregulated activation of the mononuclear phagocyte system, hyper-inflammatory response (Mehta et al., 2020), and MAS (Schulert and Grom, 2015) which ultimately leads to the death of patients.

Apart from macrophages, the activation of neutrophils has also been associated with ARDS and cytokine storm in COVID-19 patients. Cytokine profiling, blood clots, and MPO activity in patients reveal infiltration of activated neutrophils in the lungs, which is indicative of early defense mechanism against SARS – CoV-2 virus. This was made evident by the presence of Neutrophils extracellular traps (NET), which are used by activated neutrophils to trap the pathogens. Excessive infiltration and activation of neutrophils (Barnes et al., 2020) are manifested in neutrophilia, which is sufficient to predict poor outcomes in COVID-19 cases. Furthermore, the neutrophil-to-lymphocyte ratio has been associated with a high-risk factor for severe disease (Zhang et al., 2020). The accumulation of neutrophils in the lungs of COVID-19 patients was associated with acute capillaritis with fibrin deposition, extravasation of neutrophils into the alveolar space, and neutrophilic mucositis (Zhang et al., 2020). These findings potentially indicate the role of activated neutrophils also in the pathogenesis of COVID-19 cases.

During the early phase of infection in COVID-19 patients, NETs activate macrophages to secrete IL-1 and TGF beta, further activating neutrophils infiltration and activation. Therefore, both macrophages and neutrophils represent potential targets for interventions. However, due to the sensitive nature of neutrophils for ROS, RNI mediated death during pneumonitis, and pulmonary fibrosis, these cells undergo death. In COVID-19 patients, these dead neutrophils are then phagocytosed by the surrounding inflammatory macrophages, which induce the release of IL-1 beta and TGF beta, inducing the anti-inflammatory programming of macrophages (Greenlee-Wacker, 2016) and Th2 programming of the pulmonary compartment. The macrophage remains active and viable for an extended period over the neutrophils and is likely to be more proficient than neutrophils in controlling SARS-CoV-2 infection. Due to this activation and lack of neutrophils, interventions targeting these cells are particularly challenging at the moment. Therefore, macrophages are a prudent target and approach to controlling the infection.

Both activation and increased infiltration of macrophages (Giamarellos-Bourboulis et al., 2020; Wang et al., 2020) and neutrophils (Morris et al., 2020; Middleton et al., 2020) during infection leads to ARDS and cytokine storm in COVID-19 patients. During SARS-CoV-2 infection, activated CD4+ T lymphocytes secrete granulocyte-macrophage colony-stimulating factor which further stimulates committed macrophage to secrete pro-inflammatory cytokines, thereby continuing the vicious cycle of the cytokine storm.

SARS‐CoV‐2 activates alveolar, splenic, and renal macrophages through angiotensin-converting enzyme 2 (ACE2) and enhances the secretion of IL‐6, TNF-α, IL‐10, and PD‐1 (Wang et al., 2020). Both ARDS and MAS contribute to the significant depletion of CD8+ CTLs which are associated with disease severity (Luo et al., 2020) in COVID-19 patients. SARS-CoV-2 infects type 2 alveolar cells, epithelial cells, and podocytes in the lungs and kidney respectively and interacts with them *via* ACE2 receptors which facilitate the attachment and entry of this virus into host cells (Hoffmann et al., 2020; Wrapp et al., 2020). ACE-2/Angiotensin-II receptors are known to activate the Sphingosine-1-phosphate receptor 1 (S-1P receptor 1) which is known to mediate IL-6 induced myopathy and fibrosis (Ohkura et al., 2017). S-1P R-1 signaling is associated with Th2/17 responses (Schroder et al., 2011; Schulze et al., 2011), hypoxia, and allergic manifestations (Saluja et al., 2017), which altogether promote Th2 bias in the infected patients. Therefore it is rational to presume that the S-1P receptor 1 signaling would enhance the pathogenesis of COVID-19 cases. Taking this into account, blocking S-1P signaling either by application of S-1P lyase (Vijayan et al., 2017) or FTY-720/Fingolimod (Penuelas-Rivas et al., 2005; Papadopoulos et al., 2010), could modulate the pathogenesis of the novel COVID-19 disease. Considering this, FTY-720 is being explored in a Phase-2 clinical trial of COVID-19 patients (NCT04280588, MRCTA, ECFAH of FMU), the results of which have not yet been published. However, due to its immunosuppressive nature, FTY-720 is expected to only lower the hyper-inflammatory response, providing symptomatic relief in COVID-19 cases but not the clearance of the SARS-CoV-2 infection (Walsh et al., 2010).

## Pre-Clinical/Animal Models for Validation of the Proposed Concept

**Figure 1:**
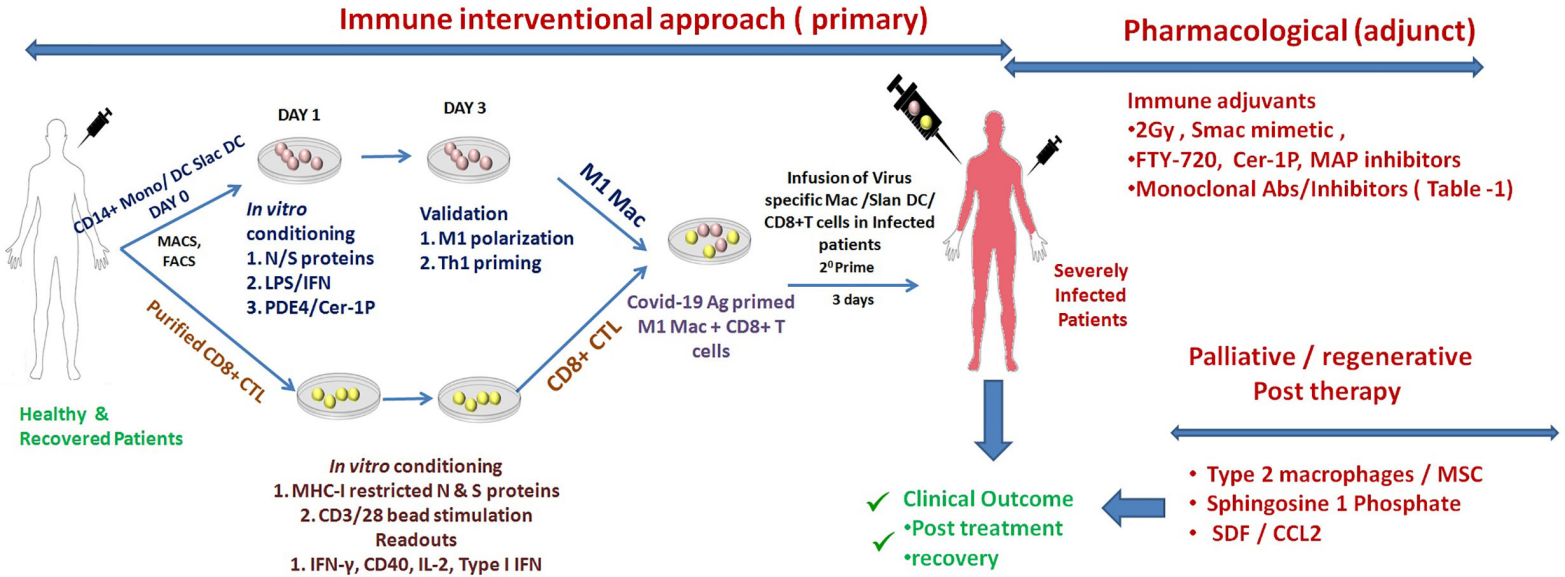
Schematic representation of augmenting SARS-Co-V2 specific adaptive immunity. Shown here are the most potent immune mediate (perspective) and/or pharmaceutical/palliative approaches for augmenting adaptive immune responses of derailed Macrophages or Slan DC for tuning effector T cell response for the treatment and recovery of COVID-19 patients. These approaches are a paramount requirement (in conjunction with other therapies) and are expected to help in managing COVID-19 disease.

Like any of the other new interventions that have been proposed since the start of the pandemic, validating a new hypothesis in a pre-clinical model is essential before its clinical application in patients. Both inbred Balb/c & C57BL/6 mice represent a suitable model of testing vaccines, antiviral drugs, and disease pathogenesis (Subbarao and Roberts, 2006). Furthermore, due to the dependency of SARS-CoV-2 on Ace-2 (Imai et al., 2005), Tmprss2 (Graham et al., 2012), and Stat-1 (Zhou P. et al., 2020) protein for manifesting disease, knockout mice of these proteins also represent potential models for studying COVID-19 disease. Most interestingly, the floxed strain of these mice could be used and backcrossed with LysM Cre mice to address macrophages related phenotype. To demonstrate the anti-COVID 19 potentials of M1 macrophages, the most suitable strategy would be to either use clinical isolate of SARS-CoV-2 or express major spike proteins of this virus in the VeroE6 cell line and co-culture them with THP/CD14+/CD11b+ macrophages to evaluate the influence of the various adjuvants (**Table 1** and **Figure 1**) on the innate and adaptive immune response of both macrophages and CD8+CTL. Several aspects like maturation, the M1/Th1 programming of macrophages, and subsequent killing of the virus or expression of Spike proteins can be used to correlate the M1 programming of RAW, THP, CD14+, and CD11b+ primary macrophages. T cell response could be measured by co-culturing T cells with infected epithelial and macrophages co-culture. Various parameters like TARC/CCL17, RANTES STAT-1, 6, calcineurin, IRF - 4, and 8 factors Th1 and Th17 response could be evaluated to address the impact of M1 programmed macrophage on T cell activation and killing off infected epithelial cells. These preclinical experiments are essential for validating the anti-COVID-19 impact of M1 and CD8+CTL. These approaches represent the most suitable ways of proving the concept at preclinical levels and need further investigation.

**Table 1:** Clinical trials targeting Activated macrophages for indicated inflammatory and tumor Diseases.

Clinical Trials	TAM Targeting agent	Target	Clinical Phase	Disease	Impact
NCT03123783	APX005M	CD40	I/II/Active	NSCLC	Macrophages activation
NCT01433172	(GM.CD40L) vaccine with CCL21	CD40	I/II/Active	Lung cancer	Targeting CD14+/CD16+ alveolar macrophages
NCT02637531	IPI-549	PI3Kγ	I/Active	Advanced solid tumor; non-small cell lung cancer; melanoma; breast cancer	Macrophage plasticity
NCT00397826	Simvastine	p38MAPK	II/Active	COPD	Macrophages activation
NCT04280588	FTY 720 (Fingolimod)	S1P	II/Active	COPD, Multiple sclerosis	proposed as a prophylactic agent against Covid19
NCT01103635	ICINCB7839	ADAM17	I/Active	Liver Cirrhosis	Dampening Kuffer cells activation
NCT04261075	IPH5201 and IPH5301	CD39 & CD73	I	Cancer	Activation of macrophages and T cells

## Translational Approach

Given the above facts, mitigating pathogenic inflammation (Th2/17 responses) and pulmonary fibrosis are paramount for the management of COVID-19 infection. In this context, our previous studies have demonstrated the potential significance of iNOS+M1 effector macrophages and CD8+CTL in controlling respiratory pathogens (Schulert and Grom, 2015; Mehta et al., 2020; Xiong et al., 2020), which are associated with community-acquired pneumonia. Based on these studies, we believe that tweaking these effector immune cells may be also sufficient to control the replication of SARS-CoV2 infection in COVID-19 cases. This could be achieved through *in situ* reprogramming of Th2 and Th17 programmed macrophages or by exchanging Th2/17 programmed macrophages with Th1 primed iNOS+CD14+M1 macrophages and CD8+CTL, which are in the neo-adjuvant setting. Based on our previous studies (Barnes et al., 2020; Zhang et al., 2020), we also believe that the exchange of Th17+ macrophages with Th1+ M1 macrophages may provide protective immunity against SARS-CoV-2 infection in cases of COVID-19. This approach could improve the prospects of severely infected patients but needs to be commissioned and explored further before clinical application.

## Proposed Intervention

While the application of antiviral drugs like Remedesvir and Tocilizumab is sufficient to control the infection, it is still questionable whether these drugs are adequate to augment immunogenic inflammation and clear infected cells from the infected organ. In this context, we propose the potential role of Ceramide-1 phosphate as immune adjuvants for promoting the Th1 programming of infected cells/tissue and subsequent clearance of virus infected cells from the tissue. Given the potential involvement of S-1P in fibrosis and Th1 bias, FTY-720 based interventions are presently under Phase-2^nd^ clinical trial (NCT04280588) based on trapping effector T cells in the lymph node for effective control of the infection and mitigating cytokine storm. However, due to its immunosuppressive nature, we believe that FTY-720 may not help patients. Other studies have also highlighted the significance of the BCG vaccination and dexamethasone on increased resistance to the infection (Escobar et al., 2020) and reduced mortality (Lammers et al., 2020) in COVID-19 patients. Since these interventions are targeted to reduce the inflammatory score and are anticipated to provide only symptomatic relief, other more robust and stable interventions are required for managing the disease.

Several of the reports mentioned above have provided compelling evidence that hyper-activated macrophages are critical for ARDS and in causing the subsequent death of patients. It is worth considering combining macrophage directed strategies with other available approaches (as adjuncts), which may be decisive for the effective management of severely infected cases. Out of several available options, the most relevant strategy would be to promote *in situ* reprogramming of Th2/17 macrophages toward M1 by employing various neo adjuvants, summarized in **Table 1**.

In the past, we have identified several neo adjuvants, such as PDE4b and AC mimetic, which are capable of modulating the Th17 response in human CD14+ monocyte and Slan DC (Oehrl et al., 2017). This reflects the results of another study (Konig et al., 2020), which highlighted the potential of androgenic receptors in modulating cytokine storm. This indicates that PDE4/AC modulators (Maier et al., 2017) could also potentially modulate and mitigate macrophage activation syndrome and help in COVID-19 cases.

Apart from these, we have validated several other immune adjuvants, such as low dose Gamma Radiation (Klug et al., 2013; Prakash et al., 2016), S-1P (Nadella et al., 2019), and Smac mimetics (Nadella et al., 2017), which indicate the potential of the Th1 programming of macrophages in a pre-clinical model system. These adjuvants are likely to create immunity against SARS-CoV-2 infection in the animal models discussed above. Various other adjuvants are included in **Table 1**, each of which targets refractory macrophages that can also potentially be utilized for tempering macrophage plasticity and could effectively control SARS-CoV-2 infection.

## Macrophages/MSC as Palliative Regimes for COVID-19

Since macrophages are a relatively plastic component of the body and could help with tissue regeneration and homeostasis after completion of therapy. These aspects are critical for the expected outcome of the proposed immune and pharmacological interventions against infection. M2 or refractory macrophages are also known to promote the activity of fibroblast and mesenchymal stem cells and fibroblasts (Yu et al., 2016; Zhang et al., 2018), which can potentially neutralize any potentially adverse impacts of therapy. Both MSC and macrophages secrete various factors (Boxman et al., 1996; Broughton et al., 2012) that are involved in wound healing. On account of the regenerative potential of macrophages (Chamoto et al., 2012; Hoegl et al., 2016) and their close association with mesenchymal stem cells (Spiller and Koh, 2017; Myneni et al., 2019), these cells can potentially be utilized in a palliative approach (Granata et al., 2010; Almatroodi et al., 2014) for the accelerated recovery of patients. This warrants the use of macrophages and MSC as whole-cell infusions or their products as palliative components for enhancing the recovery of COVID-19 patients after therapeutic interventions.

## Major Perspective

The proposed interventions depicted in **Figure 1** are candid approaches to tuning pathogenic inflammation, including the Th1 reprogramming of derailed Macrophages and enhancing the MHC-I dependent presentation of the viral antigens CD8+ CTL. These approaches provide potential means of augmenting the adaptive immunity of the host in fighting SARS-CoV-2 infection: a novel immunotherapeutic approach that is translationally viable. The approach harbors the potential for the effective management of SARS-CoV-2 infection in severely infected COVID-19 patients. We anticipate that it could be employed as a potential adjunct to placid/antiviral therapies in effectively managing COVID-19 disease, both prospective and therapeutic, in patients and which requires immediate further investigation for its clinical efficacy.

## Author Contributions

HP conceived the idea and supervised the study. DT, AJ, SK, HM, and VS contributed to the research tool. DT and VT undertook the critical analysis. HP and AJ wrote the manuscript. All authors contributed to the article and approved the submitted version.

## Conflict of Interest

The authors declare that the research was conducted in the absence of any commercial or financial relationships that could be construed as a potential conflict of interest.
